# Action myoclonus-renal failure syndrome: diagnostic applications of activity-based probes and lipid analysis

**DOI:** 10.1194/jlr.M043802

**Published:** 2014-01

**Authors:** Paulo Gaspar, Wouter W. Kallemeijn, Anneke Strijland, Saskia Scheij, Marco Van Eijk, Jan Aten, Herman S. Overkleeft, Andrea Balreira, Friederike Zunke, Michael Schwake, Clara Sá Miranda, Johannes M. F. G. Aerts

**Affiliations:** * Lysosome and Peroxisome Biology Unit (UniLiPe), Institute of Molecular and Cell Biology (IBMC), University of Oporto, Oporto, Portugal; † Biomedical Science Institute Abel Salazar (ICBAS), University of Oporto, Oporto, Portugal; § Departments of Medical Biochemistry Academic Medical Center, Amsterdam, The Netherlands; ** Pathology, Academic Medical Center, Amsterdam, The Netherlands; †† Department of Bioorganic Chemistry, University of Leiden, The Netherlands; §§ Department of Biochemistry, Christian Albrechts Universitat Kiel, Kiel, Germany; *** Department of Biochemistry, University of Bielefeld, Bielefeld, Germany

**Keywords:** Gaucher disease, macrophages/monocytes, scavenger receptors, sphingolipids, storage diseases, glucocerebrosidase, glucosylsphingosine, LIMP2/SCARB2

## Abstract

Lysosomal integral membrane protein-2 (LIMP2) mediates trafficking of glucocerebrosidase (GBA) to lysosomes. Deficiency of LIMP2 causes action myoclonus-renal failure syndrome (AMRF). LIMP2-deficient fibroblasts virtually lack GBA like the cells of patients with Gaucher disease (GD), a lysosomal storage disorder caused by mutations in the GBA gene. While GD is characterized by the presence of glucosylceramide-laden macrophages, AMRF patients do not show these. We studied the fate of GBA in relation to LIMP2 deficiency by employing recently designed activity-based probes labeling active GBA molecules. We demonstrate that GBA is almost absent in lysosomes of AMRF fibroblasts. However, white blood cells contain considerable amounts of residual enzyme. Consequently, AMRF patients do not acquire lipid-laden macrophages and do not show increased plasma levels of macrophage markers, such as chitotriosidase, in contrast to GD patients. We next investigated the consequences of LIMP2 deficiency with respect to plasma glycosphingolipid levels. Plasma glucosylceramide concentration was normal in the AMRF patients investigated as well as in LIMP2-deficient mice. However, a marked increase in the sphingoid base, glucosylsphingosine, was observed in AMRF patients and LIMP2-deficient mice. Our results suggest that combined measurements of chitotriosidase and glucosylsphingosine can be used for convenient differential laboratory diagnosis of GD and AMRF.

Glucocerebrosidase (GBA), an acid β-glucosidase encoded by the GBA gene at locus 1q21, catalyzes the lysosomal degradation of glucosylceramide into ceramide and glucose (1). Gaucher disease (GD) is caused by mutations in the GBA gene. GD is clinically very heterogeneous, ranging from severe neonatal variants to very mild variants with onset at old age. The most common phenotype is the nonneuronopathic type 1 (MIM*230800), showing almost exclusive storage of glucosylceramide in tissue macrophages, the so-called “Gaucher cells” (1, 2). These lipid-laden macrophages accumulate in various tissues, such as spleen, liver, bone marrow and lung, resulting in clinical symptoms of which cytopenia, hepatosplenomegaly, and skeletal abnormalities are the most prominent (1). Gaucher cells secrete a variety of cytokines and hydrolases (3–5). A several hundred-fold elevated level of chitotriosidase is the biochemical hallmark of GD (3). Another biochemical characteristic of GD is the several hundred-fold increased plasma concentration of the sphingoid base glucosylsphingosine (6). The sphingoid base is likely formed from accumulating glucosylceramide in GBA-deficient cells.

Being a low-abundant protein, GBA in leukocytes is difficult to detect with antibodies (7). To study GBA more closely in leukocytes, we employed novel activity-based labeling probes (ABPs) that allow ultra-sensitive visualization of active GBA molecules (8–11). Epoxides like conduritol B epoxide (CBE) and cyclophellitol form a covalent bond with the nucleophile E340 in GBA. Linking a BODIPY moiety to the C6 of cyclophellitol renders an even more potent irreversible ABP of GBA. Different ABPs have been designed by variation of the type of BODIPY (MDW941, Inhibody Red; and MDW933, Inhibody Green). The ABPs spontaneously cross membranes and allow very sensitive labeling of active GBA enzyme molecules in living cells (8).

The transport of newly formed GBA to lysosomes has been an enigma for a long time. GBA is known not to acquire mannose-6-phosphate moieties (12–15). The enzyme is not deficient in fibroblasts of mucolipidosis II and III patients suffering from defects in the formation of mannose-6-phosphate recognition signals (12). GBA was found to become membrane bound in the endoplasmic reticulum by interaction with an unknown protein (13, 14). Only a few years ago, the receptor protein was identified as lysosomal integral membrane protein-2 (LIMP2), one of the integral membrane proteins of lysosomes (16). Next, mutations in the scavenger receptor class B member 2 (SCARB2) gene, encoding LIMP2 protein, were found to cause action myoclonus-renal failure syndrome (AMRF) (MIM*602257), a fatal recessively inherited disorder characterized by glomerulosclerosis, progressive myoclonus epilepsy, ataxia, and accumulation of undefined storage material in the brain (17–23). More recently it has become clear that not all AMRF patients develop renal complications (22, 23). Of interest, AMRF patients do not show the massive occurrence of lipid-laden macrophages and similar pathology to GD patients (24). Consistently, AMRF patients also do not show elevated plasma chitotriosidase. These findings point to cell-type-specific consequences of LIMP2 deficiency. Indeed, LIMP2-deficient fibroblasts of AMRF patients lack GBA, whereas this appears not to be the case for their white blood cells (18).

The availability of ABPs allowing sensitive detection of GBA in leukocytes prompted us to study the fate of the enzyme in LIMP2-deficient cells, obtained from an AMRF patient as well as from LIMP2-deficient mice. We here report the outcome of these investigations, confirming cell-type-specific reductions in GBA caused by LIMP2 deficiency. Furthermore, we studied the consequences of LIMP2 deficiency for glucosylceramide and glucosylsphingosine levels, the products of hydrolysis by GBA. We here report the outcome and possible use in the differential diagnosis of AMRF and GD.

## MATERIALS AND METHODS

### ABPs

MDW941 (Inhibody Red) and MDW933 (Inhibody Green) were synthesized as described earlier (8).

### Antibodies

The rabbit polyclonal anti-LIMP2 antibody was purchased to Novus Biologicals, Littleton, CO.

### AMRF patient materials

Materials from donors were obtained after informed consent. Leukocytes and fibroblasts were obtained from LIMP2-deficient patients homozygous for the SCARB2/LIMP2 mutation W178X (18), LIMP2 obligate carriers of the same mutation, GD patients with the GBA genotype L444P/L444P, and control subjects. Fibroblasts obtained from skin biopsies were cultured in Dulbecco's modified Eagle's medium supplemented with 10% fetal bovine serum, 2 mM l-glutamine, 1% penicillin-streptomycin, 100 mg/ml kanamycin sulfate, and 2.5 mg/ml Fungizone (Gibco, Invitrogen). Cells were harvested when confluence was reached. Leukocytes were isolated from whole blood as described earlier (18).

Monocytes were isolated with CD14 MicroBeads (Miltenyi Biotec) according to the manufacturer's protocol. Isolated monocytes were differentiated into macrophages during 7 days in RPMI medium supplemented with 10% human AB serum (3).

### Mice

Mice were housed and plasma was collected according to the local protocol with approval from the review board of the Christian-Albrechts-University in Kiel (Germany). LIMP2-deficient mice were generated as described in (16).

### Preparation of cell lysates

Isolated fibroblasts, leukocytes, and macrophages were homogenized in potassium phosphate buffer [25 mM (pH 6.5), 0.1% (v/v) Triton X-100]. Protein was quantified by BCA kit (Thermo Scientific).

### Labeling of GBA with ABPs

Cell homogenates were incubated with MDW941 (100 nM) in McIlvaine buffer [150 mM citrate-Na_2_HPO_4_ (pH 5.2), 0.2% (w/v) sodium taurocholate, 0.1% (v/v) Triton X-100] for 30 min at 37°C and subjected to SDS-PAGE. Fluorescent GBA on slab gels was visualized using a fluorescence scanner (Typhoon variable mode imager, Amersham Biosciences) (8).

Intact fibroblasts were incubated with MDW941 (5 nM) for 72 h, and subsequently the culture medium was collected and the cells were harvested. Detection of fluorescent GBA in the culture medium was performed upon capture of the enzyme from 1 ml medium using monoclonal antibody 8E4 immobilized to Sepharose beads (25).

For fluorescence-activated cell sorting (FACS), fibroblasts and macrophages were incubated with MDW933 (50 nM) for 5 h in the medium (8). In the case of white blood cells, leukocytes were collected from freshly drawn blood washed with 0.8% (w/v) ammonium chloride solution and lysing the remaining erythrocytes. Leukocytes were next incubated with MDW933 (100 nM) for 30 min in phosphate buffered saline containing 1% (w/v) BSA. FACS analysis was performed with a FACSCalibur (B.D. Bioscience), λ_ex_ 488 nm, λ_em_ 530 nm (bandpass filter 30 nm) (8).

### Western blotting

SDS-PAGE gels were electroblotted onto a nitrocellulose membrane (Schleicher and Schuell). Membranes were blocked with 5% skimmed milk and 0.05% Tween-20 in Tris-buffered saline (TBS) for 1 h at room temperature and incubated overnight with the primary antibodies at 4°C. Membranes were then washed three times with 0.01% Tween-20 in TBS and incubated with the appropriate IRDye conjugated secondary antibodies for 1 h at room temperature. After washing, detection was performed using the Odyssey® CLx infrared imaging system.

### Measurement of in vivo GBA enzymatic activity

GBA enzymatic activity in intact fibroblasts, leukocytes, and macrophages was measured using 5-(pentafluorobenzoylamino)fluorescein di-β-d-glucopyranoside (PFB-FDG) (26) (20 μM for fibroblasts, 40 μM for leukocytes and macrophages) as substrate, exactly as described by Witte et al. (8). To discriminate the activity of lysosomal GBA from other β-glucosidases, samples were pretreated with and without 1 mM of CBE for 30 min before adding the PFB-FDG. No activity was observed for fibroblasts preincubated with CBE, indicating that activity in the absence of the inhibitor is due to GBA.

### Fluorescence microscopy

Fibroblasts were cultured on glass slides. Cells were incubated with MDW933 (50 nM) for 2 h in medium. Next, cells were washed, fixed with 3% (v/v) paraformaldehyde in PBS for 15 min, washed, incubated with 0.1 mM NH_4_Cl in PBS for 10 min, and then incubated with 3% (w/v) BSA and 10% (w/v) goat serum albumin in PBS for 1 h. Nuclei were stained with 4’,6-diamidino-2-phenylindole (DAPI). The filter blocks used were A4 (360/40 nm band pass excitation, 400 nm dichromatic mirror, 470/40 nm band pass suppression) for DAPI and K3 (470–490 nm band pass excitation, 510 nm dichromatic mirror, 515 nm long pass suppression) for MDW933. Analysis was performed with multispectral imaging, as described before (8).

### Glucosylceramide and glucosylsphingosine measurements

Glucosylceramide and glucosylsphingosine in fibroblasts, leukocytes, and plasma specimens were measured as described earlier in (27) and (6), respectively.

### Statistical analysis

The results were analyzed using the Student's unpaired *t*-test. *P* < 0.05 was considered significant. Data were statistically analyzed using GraphPad Prism 6 software (Graphpad Software, San Diego, CA).

## RESULTS

### Detection of active GBA in fibroblasts, leukocytes, and macrophages

**Fig. 1.**
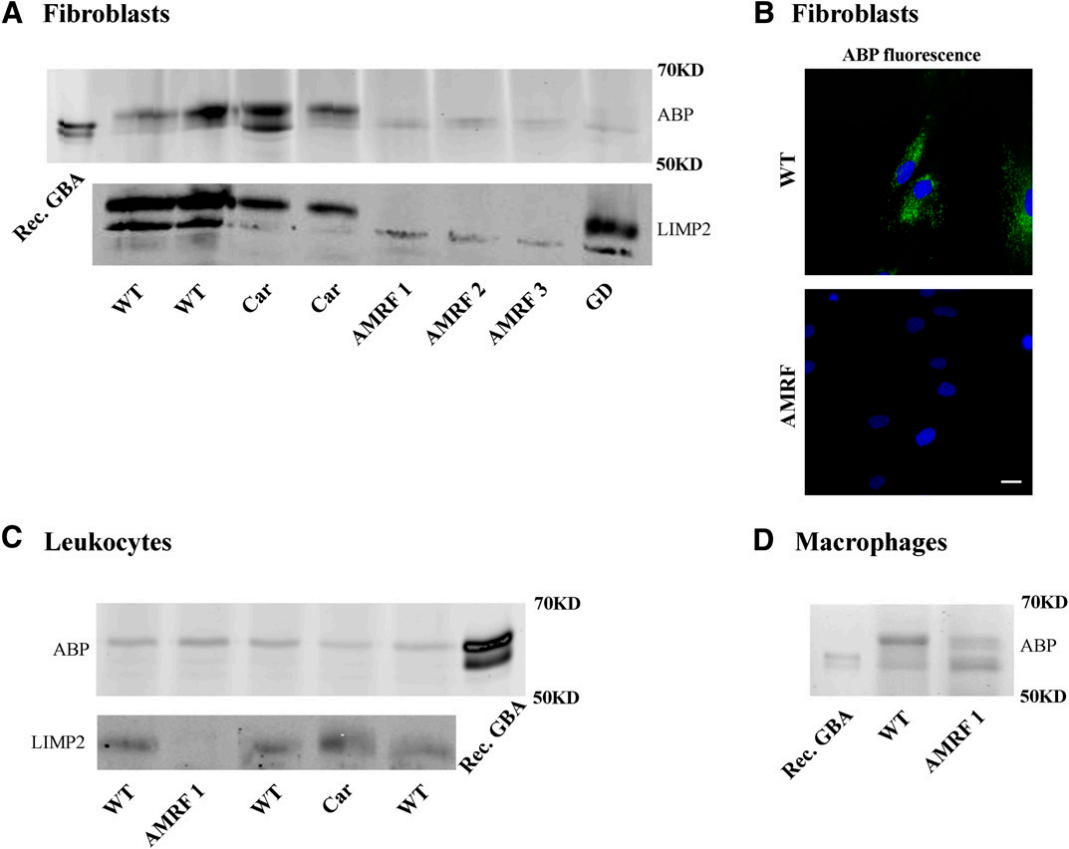
Visualization of GBA with fluorescent ABPs MDW941 and MDW933. A: Detection of GBA in homogenates of cultured fibroblasts (30 μg): labeling followed by SDS-PAGE and detection by fluorescence imaging. AMRF 1, patient 1 (LIMP2 W178X/W178X); AMRF 2, patient 2 (LIMP2 W178X/W178X); AMRF 3, patient 3 (LIMP2 W178X/W178X); Car, carrier of AMRF (LIMP2 W178X/WT); GD, type 2 GD patient; Rec. GBA, recombinant GBA (Cerezyme). B: Fluorescence microscopy. Fibroblasts labeled in vivo with MDW933 and DAPI [Upper micrograph, control fibroblasts (WT); lower micrograph, AMRF patient]. The scale bar represents 20 μm. C: Detection of GBA in homogenates of leukocytes (50 μg): labeling followed by SDS-PAGE and detection by fluorescence imaging. D: Detection of GBA in homogenates of cultured macrophages (20 μg): labeling followed by SDS-PAGE and detection by fluorescence imaging.

To detect active GBA molecules, homogenates of fibroblasts from three AMRF patients (LIMP2 W178X/W178X), two AMRF carriers (LIMP2 W178X/WT), two control subjects, and one type 2 GD patient (GBA L444P/L444P) were labeled with MDW941 (Inhibody Red) and subjected to SDS-PAGE. We demonstrated earlier that MDW941 specifically labels lysosomal GBA and no other retaining β-glucosidases in humans (GBA2 and GBA3) (8, 28). Actually, cultured fibroblasts contain no GBA3 and very little GBA2 (29). GBA, labeled after incubation of fibroblasts with MDW941, was detected by fluorescence imaging of the slab gel (**Fig. 1A**). Fibroblasts of LIMP2-deficient AMRF patients showed almost no active GBA. The active GBA was similarly reduced in cells from the type 2 GD patient. In the case of cells from the AMRF carrier, a normal amount of active GBA was detected.

Next, intact fibroblasts were incubated with MDW933 to visualize the active GBA molecules by fluorescence microscopy (Fig. 1B). Multi-spectral imaging was used to differentiate autofluorescence from true ABP signals. In line with the results in Fig. 1A, AMRF fibroblasts were found to be clearly deficient in lysosomal GBA compared with the WT.

The findings with leukocytes of an AMRF patient were very different from those of fibroblasts. Labeled active GBA in patient leukocytes was almost similar when compared with cells from a healthy subject (Fig. 1C).

Western blot analysis using anti-LIMP2 antibody showed that LIMP2 protein was expressed at higher levels in fibroblasts compared with leukocytes. LIMP2 was decreased in fibroblasts of AMRF carriers compared with controls (Fig. 1A, C). Next, GBA was analyzed in monocyte-derived macrophages. Monocytes were isolated from the blood of an AMRF patient and a healthy subject and differentiated into macrophages. Detection of active GBA in homogenates of the generated macrophages revealed again only a slight reduction of GBA in the patient's cells (Fig. 1D).

**Fig. 2.**
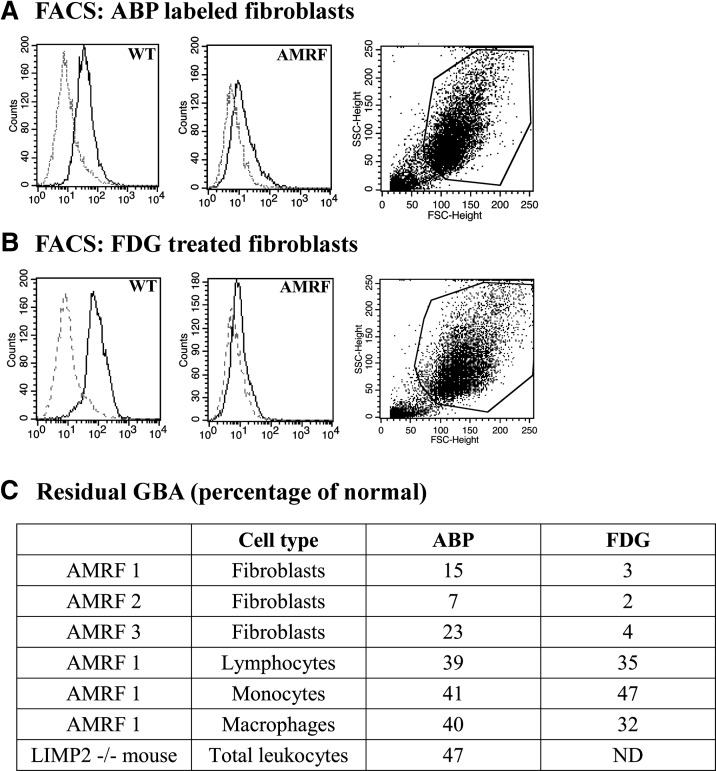
FACS analysis of active GBA detected with ABP MDW933 and PFB-FDG as substrate. A: Example of FACS analysis of fibroblasts with an ABP. Left and middle panels: primary FACS data (dotted line, not labeled with MDW933; solid line, labeled with MDW933). Right panel: representative FACS dot plot. B: Example of FACS analysis of fibroblast enzymatic activity toward PFB-FDG. Panels as in (A). C: Overview of detected residual active GBA by analysis of cells with an ABP and PFB-FDG.

Next, the amount of active GBA in intact cells was assessed by FACS analysis. Cells were labeled with ABP MDW933, or their GBA activity was determined by incubation with the substrate PFB-FDG (**Fig. 2**). In fibroblasts of three AMRF patients, active GBA was found to be reduced using ABP labeling (Fig. 2A). We also used the cell permeable substrate PFB-FDG for assessing GBA enzymatic activity (Fig. 2B). We checked to ascertain whether all detected activity could be ascribed to lysosomal GBA by preincubation with and without CBE, an irreversible inhibitor (data not shown).

Fibroblasts of an AMRF carrier showed considerable levels of active GBA. Similar experiments showed that the amount of active GBA was relatively high in the blood cells of an AMRF patient (Fig. 2C). Significant residual GBA levels (values >30%) were noted in lymphocytes, monocytes, and cultured macrophages of the AMRF patient, both with ABP labeling and PFB-FDG treatment. The same finding was made for total leukocytes from LIMP2-deficient mice (Fig. 2C). Measurement of GBA enzymatic activity in cell lysates using the artificial substrate 4-methylumbelliferyl-β-d-glucoside (30) gave similar results (data not shown).

### Secretion of GBA by LIMP2-deficient fibroblasts

**Fig. 3.**
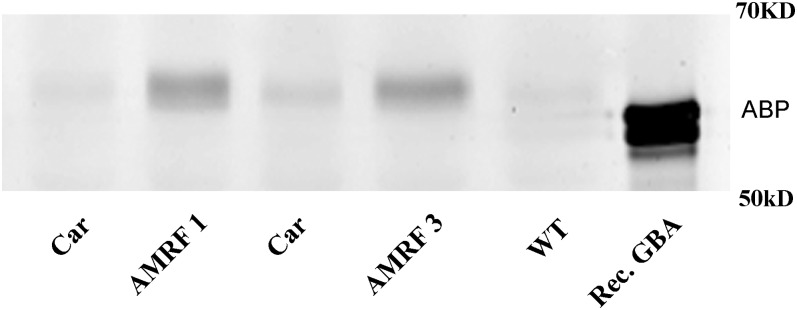
Partial secretion of GBA by fibroblasts. Fibroblasts were labeled for 72 h with MDW941. From 1 ml medium, GBA was immunoprecipitated with anti-GBA monoclonal antibody (8E4) immobilized to Sepharose beads. The immunoprecipitate, subjected to SDS-PAGE and fluorescent GBA labeled by MDW941, was visualized by imaging. Lane 1, carrier (Car) of AMRF (LIMP2 W178X/WT); lane 2, AMRF patient 1 (AMRF 1) (LIMP2 W178X/W178X); lane 3, carrier of AMRF (LIMP2 W178X/WT); lane 4, AMRF patient 3 (AMRF 3) (LIMP2 W178X/W178X); lane 5, control subject (WT) (LIMP2 WT); lane 6, recombinant GBA (Rec. GBA).

Misrouting of GBA due to absence of LIMP2 might result in partial secretion of the enzyme to the extracellular space. To study this, cultured fibroblasts were labeled with MDW941 for 72 h and the culture medium was collected. GBA from the medium (1 ml) was immunoprecipitated with the anti-GBA monoclonal antibody (8E4) and visualized by fluorescence scanning of SDS-PAGE gels (**Fig. 3**). Indeed, fibroblasts of AMRF patients demonstrated the presence of ABP-labeled GBA in the medium, which was absent in the WT and AMRF carriers. Of note, the molecular mass of GBA seen with SDS-PAGE is determined by its N-linked glycan composition. The enzyme was initially synthesized with four high-mannose type glycans showing a mass of about 62 kDa with SDS-PAGE. In the Golgi apparatus, at least three glycans were modified to sialylated complex type structures rendering masses of 62–66 kDa. After entering the lysosome, the glycans are trimmed by local exoglycosidases resulting in a gradual reduction to 59 kDa (31). Secreted enzyme largely shows a high molecular mass due to the presence of complex-type glycans, lacking the lysosomal deglycosylation. We presume that the very tiny amounts of lower mass bands, seen in the case of control fibroblasts but not AMRF fibroblasts, stem from lysosomal extrusion/secretion.

We next looked for the presence of GBA in the plasma of AMRF patients by measuring its enzymatic activity with the artificial substrate 4-methylumbelliferyl-β-d-glucoside (30). We noted an increased activity (10.3 vs. 1.4 nmol of released 4-methylumbelliferone per milliliter of plasma per hour) in the case of fresh specimens from an AMRF patient versus a healthy subject. Importantly, the increased activity was only detected in freshly obtained plasma. Storage of plasma, even frozen, led to inactivation of the enzymatic activity of GBA; in samples frozen for a long period at −20°C, less than 2% of the original GBA activity was detected. In line with the data for freshly obtained AMRF patient plasma, LIMP2-deficient mice showed increased GBA as well (167 nmol/ml of plasma per hour vs. 22 nmol/ml of plasma per hour for WT mice). Given the loss of enzyme activity in plasma, measurement of plasma GBA activity is in practice not a very reliable test to identify AMRF patients.

### Lipid abnormalities in relation to LIMP2 deficiency

The lack of lysosomal GBA as the result of LIMP2 deficiency might theoretically result in abnormalities in glucosylceramide breakdown as seen in GD patients. We earlier documented the increase of glucosylsphingosine in plasma of symptomatic type 1 GD patients suffering from a primary defect in lysosomal GBA (6). Because LIMP2 deficiency also results in a marked reduction of lysosomal GBA, we assumed that the same would occur in AMRF patients.

We therefore determined glucosylceramide and glucosylsphingosine concentrations in the fibroblasts and plasma of AMRF patients as well as in the plasma of LIMP2-deficient mice.

**Fig. 4.**
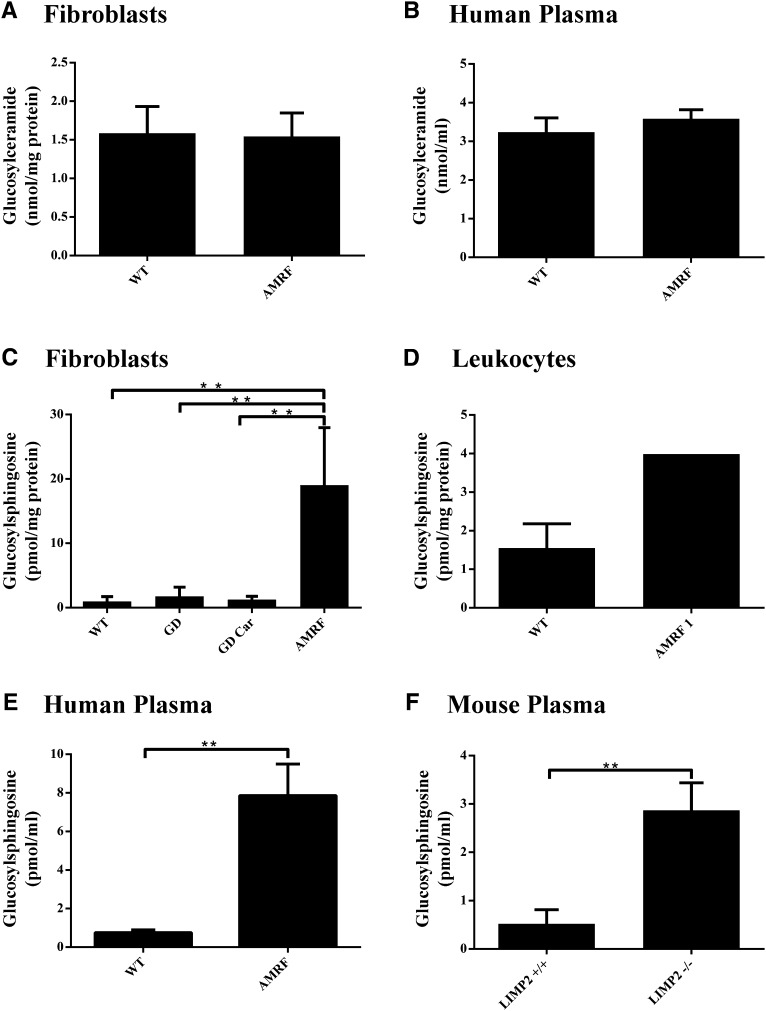
Glucosylceramide and glucosylsphingosine content of cells and plasma in relation to AMRF/LIMP2 deficiency. Glucosylceramide and glucosylsphingosine were determined as described in Materials and Methods. A: Glucosylceramide (nmol/mg total protein) in AMRF fibroblasts (n = 2). B: Glucosylceramide (nmol/ml total protein) in human AMRF plasma specimens (n = 2). C: Glucosylsphingosine (pmol/mg total protein) in AMRF fibroblasts (n = 3). D: Glucosylsphingosine (pmol/mg total protein) in AMRF leukocytes (n = 1). E: Glucosylsphingosine (pmol/ml) in human AMRF plasma specimens (n = 3). F: Glucosylsphingosine (pmol/ml) in samples from LIMP2^−/−^ mice (n = 3). AMRF, LIMP2-deficient patients (LIMP2 W178X/W178X); GD, GD patients; GD Car, GD carriers; LIMP2^+/+^, WT mice; LIMP2^−/−^, KO LIMP2 mice. **P < 0.01, unpaired student's *t*-test).

In AMRF fibroblasts, glucosylceramide was not elevated compared with the control range (**Fig. 4A**). Importantly, a marked increase in glucosylsphingosine was noted (Fig. 4C). The same, although not to the same extent of elevation, was observed for leukocytes (Fig. 4D). We also detected elevated glucosylsphingosine levels in the plasma of three AMRF patients (Fig. 4E). No clear concomitant increase in glucosylceramide was observed as compared with the matched control (Fig. 4B). To further validate the finding of the sphingoid base abnormality, we also examined plasma of LIMP2-deficient mice. Again, a clear elevation in glucosylsphingosine concentration was observed (Fig. 4F).

## DISCUSSION

The study by Reczek et al. (16) first revealed that the lysosomal integral membrane protein, LIMP2, mediates trafficking of newly formed GBA to lysosomes. Soon afterwards Berkovic et al. (17) demonstrated that LIMP2 deficiency due to mutations in the SCARB2 gene forms the basis for AMRF. Independently, Balreira et al. (18) described that mutations in the SCARB2 gene cause cell-type-specific GBA deficiency. Intriguingly, AMRF patients do not show the lipid-laden macrophages that characteristically occur in GD patients suffering from a primary defect in GBA. In GBA-deficient GD patients, the macrophages are particularly prone to store glucosylceramide and to transform to so-called Gaucher cells (32). Gaucher cells produce and secrete unique marker proteins such as chitotriosidase and CCL18 (32). Characteristically, chitotriosidase is several hundred-fold higher in the plasma of symptomatic GD patients (3, 33). Of interest, AMRF patients do not show elevated levels of chitotriosidase like GD patients (18, 24) (P. Gaspar and C. Sá Miranda, unpublished observations). This suggests that somehow macrophages in LIMP2-deficient individuals can still adequately degrade glucosylceramide due to the presence of sufficient GBA in their lysosomes. We demonstrate here with a variety of techniques that, indeed, white blood cells of AMRF patients have considerable residual GBA, in sharp contrast to fibroblasts. We employed newly designed ABPs that allow fluorescent labeling of active GBA molecules. We first showed that much less active GBA can be detected in homogenates of AMRF fibroblasts with an ABP. In white blood cells and cultured macrophages, this abnormality is far less striking. Next we labeled intact cells with an ABP and noted by FACS analysis that AMRF fibroblasts are markedly deficient in active GBA. Significant residual active GBA was again detected in lymphocytes, monocytes, and cultured macrophages. The same phenomenon was demonstrated in an independent manner by exploiting PFB-FDG as substrate for in vivo detection of GBA enzymatic activity. Again, FACS analysis revealed a marked deficiency in AMRF fibroblasts, but considerable residual enzyme in white blood cell types. We next looked into the fate of GBA in fibroblasts of AMRF patients, observing that these cells abnormally secrete some active GBA to the medium where the enzyme rapidly loses its enzymatic activity. Labile GBA activity is demonstrable in freshly obtained plasma of AMRF patients. The mechanism by which lysosomes of white blood cells acquire sufficient GBA is presently not known and a topic of further investigation. It can't be excluded that these cells possess a membrane protein other than LIMP2 that governs intracellular transport of GBA from the endoplasmic reticulum to lysosomes. Alternatively, secreted GBA might be taken up by some endocytotic process and delivered via this secretion-recapture manner to lysosomes of white blood cells.

In lysosomes, assisted by the activator protein saposin C, GBA takes care of degradation of glucosylceramide (4). Deficiency of GBA causes formation of glucosylceramide storage tubules, most prominent in macrophages. Of interest, part of the accumulating glucosylceramide during GBA deficiency is deacylated to the sphingoid base, glucosylsphingosine, which can leave lysosomes and cells (6). Most likely, acid ceramidase is responsible for glucosylsphingosine formation, because acid ceramidase-deficient Farber disease fibroblasts have been found to be unable to synthesize glucosylsphingosine upon inhibition of GBA activity (34).

This explains the marked increase in glucosylsphingosine in the plasma of GD patients. Our investigation of glucosylceramide and glucosylsphingosine concentrations in cultured fibroblasts of AMRF patients revealed that only the sphingoid base, glucosylsphingosine, is markedly increased. Consistent with this, glucosylsphingosine is abnormally high in the plasma of AMRF patients. This finding was also made with plasma specimens of LIMP2-deficient mice. Apparently, in AMRF patients, cells other than macrophages also form glucosylsphingosine during GBA deficiency that is partly released into the circulation.

Our investigation renders a workflow for convenient laboratory diagnosis of AMRF prior to sequencing of the SCARB2 gene. If markedly increased plasma glucosylsphingosine is detected in an individual in the absence of elevated chitotriosidase, this is an indication for AMRF. Measurement of GBA activity in white blood cells will not be very informative, but in fibroblasts enzymatic activity needs to be reduced to consider further the diagnosis AMRF. Such a diagnosis can be further substantiated with the sequencing of the SCARB2 gene. Demonstration of functional GBA deficiency by detection of abnormally high glucosylsphingosine in plasma should be considered as an important step in identification of individuals suffering from a truly functional deficiency in LIMP2.
